# Associations of Free Triiodothyronine With Sarcopenia Among Patients With Type 2 Diabetes: Cross‐Sectional and Sex‐Stratified Analyses

**DOI:** 10.1155/jdr/3134701

**Published:** 2026-02-08

**Authors:** Jing Zhao, Mei-Tong Zhang, Dan Yu, Zi-Yue Shao, Da-Shuang Chen, Jian Zhu

**Affiliations:** ^1^ Department of Endocrinology, Affiliated Hospital of Jiangnan University, Jiangnan University, Wuxi, Jiangsu, China, jiangnan.edu.cn; ^2^ Department of Endocrinology, Nanjing First Hospital, Nanjing Medical University, Nanjing, China, njmu.edu.cn

**Keywords:** Asian Working Group for 2019, free triiodothyronine, sarcopenia, thyroid hormones, Type 2 diabetes mellitus

## Abstract

**Objective:**

Sarcopenia is prevalent in Type 2 diabetes mellitus (T2DM). Whether thyroid‐related hormones within the euthyroid range can help identify sarcopenia risk remains unclear. This study was aimed at evaluating the association of euthyroid‐range thyroid markers with sarcopenia and their utility in risk stratification among adults with T2DM.

**Methods:**

We analyzed 1823 adults with T2DM (2019–2023). Sarcopenia was defined per Asian Working Group for Sarcopenia 2019 criteria. Muscle mass was assessed via multifrequency bioelectrical impedance analysis, handgrip strength via dynamometer, and gait speed via 6‐m walk test. Multivariate linear and logistic models were adjusted for demographics, diabetes duration, body mass index, nephropathy, glycated hemoglobin, and antidiabetic medications. Discriminatory ability was evaluated using receiver operating characteristic curves, change in the area under the receiver operating characteristic curve (*Δ*AUROC), and net reclassification indices (net reclassification improvement [NRI]; bootstrapped).

**Results:**

Higher free triiodothyronine (FT3) levels correlated with greater muscle mass, handgrip strength, and gait speed. Among thyroid markers, FT3 showed the strongest discrimination for sarcopenia (AUROC = 0.633). The optimal cutoff was 3.62 pmol/L (sensitivity, 85.6% and specificity, 35.7%), although overall performance was modest. Low FT3 independently predicted sarcopenia (odds ratio [OR], 2.26; *p* = 0.002). The association remained significant in females (OR, 3.29) but not in males (OR, 1.83); no sex interaction was detected. Adding FT3 modestly improved the adjusted model (*Δ*AUROC, 0.007; NRI significant at 25th percentile risk).

**Conclusion:**

FT3 provides modest but superior discrimination than thyroxine or thyroid‐stimulating hormone and may support early sarcopenia risk detection in T2DM, particularly at low levels, with a possible sex‐specific pattern but no significant interaction.

## 1. Introduction

Type 2 diabetes mellitus (T2DM) affects over 500 million adults globally and is projected to reach approximately 800 million by 2045 [1]. Sarcopenia, the progressive loss of muscle mass and function, develops earlier and progresses faster in individuals with diabetes than in those without; meta‐analyses confirm higher prevalence and risk [2–6]. It contributes not only to frailty and disability but also to falls, fractures, cardiovascular complications, and increased mortality [6–10]. Coexisting sarcopenia and osteoporosis further elevate fracture risk [9, 10], reinforcing the need for early detection in patients with T2DM.

Thyroid hormones are central to energy metabolism and skeletal muscle regulation [11]. Altered thyroid function correlates with changes in body composition, muscle strength, and mobility [12, 13]. In euthyroid populations, findings are inconsistent: Some studies link lower free triiodothyronine (FT3) level or reduced FT3‐to‐free thyroxine (FT4) ratio to decreased muscle mass or higher sarcopenia risk [13–16]; others report no association [17]. Among key thyroid markers (FT3, FT4, and thyroid‐stimulating hormone [TSH]), emerging evidence suggests that FT3 may be most relevant to muscle health [13–15], though comparative analyses across markers remain limited.

Evidence is even scarcer in T2DM cohorts. A few small‐scale Asian studies have suggested associations between low FT3 or low FT3/FT4 ratio and sarcopenia [18–21], but sample sizes were limited and findings inconsistent. Biological sex differences (in thyroid physiology, body composition, and hormonal milieu) may drive sex‐specific sarcopenia risk patterns. However, few studies have formally tested sex‐stratified associations between thyroid hormone levels and sarcopenia in T2DM [20, 21]. This represents an important gap, as understanding sex‐specific effects could refine risk stratification and personalized interventions.

Beyond identifying the associations, it remains unclear whether thyroid markers significantly enhance sarcopenia risk prediction in T2DM. No large studies have evaluated FT3′s incremental predictive value using statistical metrics such as the area under the receiver operating characteristic curve (AUROC), net reclassification improvement (NRI), and integrated discrimination improvement (IDI) [15–17]. Addressing this gap is crucial because biomarkers with predictive utility can enhance clinical screening.

Therefore, in this cross‐sectional study, we aimed to systematically evaluate the association of FT3, FT4, and TSH levels with sarcopenia (per Asian Working Group for Sarcopenia 2019 criteria) [22]. Specifically, we examined the following: (1) whether FT3 is independently associated with sarcopenia and muscle function; (2) whether these associations differ by sex; and (3) whether FT3 adds predictive value for sarcopenia risk beyond established risk factors.

## 2. Materials and Methods

### 2.1. Study Participants

This retrospective study initially screened 2086 patients with T2DM admitted to the Department of Endocrinology at Nanjing First Hospital between May 2019 and October 2023. Eligible participants were aged ≥ 45 years and met the diagnostic criteria of the American Diabetes Association [23], defined as follows: (1) glycated hemoglobin (HbA1c) ≥ 6.5*%*; (2) fasting plasma glucose ≥ 7.0 mmol/L; (3) a prior clinical diagnosis of diabetes; or (4) current use of antidiabetic medications. The exclusion criteria were as follows: (1) severe cardiac, hepatic, or renal disease, or major complications (e.g., cancer and cerebrovascular events); (2) history of thyroid surgery, subacute thyroiditis, or confirmed thyroid disorders (hyperthyroidism, hypothyroidism, or Hashimoto′s thyroiditis; *n* = 42); (3) acute infection or inflammation; and (4) incomplete clinical or laboratory data (*n* = 221).

After exclusions, 1823 patients (1110 men and 713 women) were included in the final analysis. Ethical approval was waived as the study used deidentified, retrospectively collected data.

### 2.2. Definitions and Measurements

Sarcopenia was defined per AWGS 2019 criteria [22]: low muscle mass plus either low muscle strength or poor physical performance. Appendicular skeletal muscle mass (ASM) was obtained from the segmental analysis of the multifrequency bioelectrical impedance device (BIA; InBody 770, InBody Co., Seoul, Korea) and as the sum of the lean mass of both arms and both legs (kilogram). The appendicular skeletal muscle mass index (ASMI) was calculated as ASM/height^2^ (kilogram per square meter), with low muscle mass defined according to AWGS 2019 cutoffs (< 7.0 kg/m^2^ for men and < 5.7 kg/m^2^ for women). Handgrip strength was measured twice per hand using a handheld dynamometer (CAMRY EH101, Xiangshan, China); maximum value was recorded, with cutoffs < 28 kg for men and < 18 kg for women. Physical performance was assessed using gait speed over a 6‐m walk at usual pace, with the faster of the two trials recorded; the cutoff value was < 1.0 m/s.

Fasting venous blood (collected in the morning and drawn within 24 h of admission) was used to measure FT3, FT4, TSH, and other biomarkers. All analyses were performed at the hospital′s central laboratory. Thyroid function indicators were determined using chemiluminescence immunoassay (reference ranges: FT3, 3.1–6.8 pmol/L; FT4, 12.0–22.0 pmol/L; and TSH, 0.27–4.20 mIU/L).

Demographic and clinical data (age, sex, smoking/alcohol use, diabetes duration, height, weight, HbA1c, and renal/hepatic function) were extracted from medical records. Additional covariates included diabetic nephropathy (urinary albumin‐to‐creatinine ratio > 30 mg/g or 24‐h urinary albumin excretion > 30 mg) and medication use (insulin and *α*‐glucosidase inhibitors). All participants had an estimated glomerular filtration rate (eGFR) above 30 mL/min/1.73 m^2^, and those receiving dialysis were excluded.

### 2.3. Statistical Analysis

Continuous variables were expressed as mean ± standard deviation or median (interquartile range), and categorical variables were expressed as frequencies (percentages). Group comparisons were performed using independent *t*‐tests or Mann–Whitney *U* test for continuous variables and *χ* ^2^ test for categorical variables. To assess linear trends across ordered FT3 categories, the Cochran–Armitage test and STATA’s “nptrend” command were applied. Statistical significance was set at *p* < 0.05.

Multivariable linear regression models were used to examine the associations between thyroid function indicators (FT3, FT4, and TSH) and continuous muscle parameters, including ASMI, handgrip strength, and 6‐m gait speed. Logistic regression models were used to evaluate the association between thyroid function and sarcopenia. Thyroid hormones were analyzed as continuous variables, and relevant categorical variables were constructed based on data‐driven cutoffs. Models were adjusted for age, sex, duration of diabetes, body mass index (BMI), HbA1c, diabetic nephropathy, and medication use (insulin and *α*‐glucosidase inhibitors).

To minimize residual confounding factors, covariates were selected based on clinical relevance and preliminary statistical testing. Univariate logistic regression analyses showed only *α*‐glucosidase inhibitors and insulin were significantly associated with sarcopenia; these were retained in fully adjusted models. Additionally, HbA1c was included as a marker of long‐term glycemic control. Final models adjusted for core confounders (age, sex, diabetes duration, BMI, and nephropathy), ensuring comprehensive yet parsimonious control.

To assess discriminative ability, receiver operating characteristic (ROC) curves and the area under the curve (AUC) were calculated. The optimal FT3 cutoff was determined using the Youden index, with corresponding sensitivity and specificity reported. Incremental predictive value beyond conventional covariates was evaluated by calculating the change in the area under the receiver operating characteristic curve (*Δ*AUROC), NRI, and IDI. Regression results were presented as *β* coefficients with standard errors for linear models and odds ratios (ORs; with 95% confidence intervals [CIs]) for logistic models. All statistical analyses were performed using STATA Version 17 (StataCorp LLC, College Station, TX, United States) for descriptive statistics, regression modeling, and trend analyses. R Version 4.2.1 (R Foundation for Statistical Computing, Vienna, Austria) was used for ROC curve analysis, incremental predictive metrics (*Δ*AUROC, NRI, and IDI), and data visualization.

## 3. Results

### 3.1. Baseline Characteristics of the Study Population

In total, 1823 patients with T2DM were included (1110 men and 713 women). Women were older than men; however, their BMI and HbA1c levels did not differ significantly. Men were more likely to smoke and consume alcohol, had higher serum creatinine and liver enzyme levels, and more often used insulin or *α*‐glucosidase inhibitors. Women had higher total cholesterol and more frequent use of metformin, sulfonylureas, and GLP‐1 receptor agonists. Thyroid function profiles also differed by sex, with men having higher FT3 and FT4 levels and women having higher TSH levels. Muscle parameters (ASMI, handgrip strength, and gait speed) were lower in women, yet sarcopenia prevalence was similar between the sexes (7.3% vs. 8.1%, *p* = 0.511) (Table 1).

**Table 1:** Clinical characteristics of study subjects in the study.

**Variables**	**Male (** **n** = 1110**)**	**Female (** **n** = 713**)**	**p** **value**
Demographic variables			
Age (years)	61.77 ± 9.47	64.38 ± 9.29	< 0.001
BMI (kg/m^2^)	24.79 ± 2.93	24.88 ± 3.41	0.534
Smoking status, *n* (%)			< 0.001
Current	415 (37.4%)	9 (1.3%)	
Past	61 (5.5%)	0	
Alcohol consumption status, *n* (%)	252	6	< 0.001
Duration of diabetes, months	120 (48–180)	120 (48–180)	0.020
Diabetic nephropathy, *n* (%)	405 (36.5%)	242 (33.9%)	0.268
Hypertension, *n* (%)	660 (59.5%)	443 (62.1%)	0.255
Sarcopenia, *n* (%)	81 (7.3%)	58 (8.1%)	0.511
Antidiabetic medication utilization, *n* (%)			
Metformin	417 (37.6%)	302 (42.4%)	0.041
Pioglitazone	24 (2.2%)	19 (2.7%)	0.490
SGLT2i	148 (13.3%)	70 (9.8%)	0.024
*α*‐Glucosidase inhibitors	268 (24.2%)	205 (28.8%)	0.029
Sulfonylureas	238 (21.4%)	202 (28.3%)	0.001
Meglitinides (repaglinide)	40 (3.6%)	33 (4.6%)	0.276
GLP‐1 RAs	36 (3.2%)	44 (6.2%)	0.003
DPP‐4i	150 (13.5%)	84 (11.8%)	0.281
Insulin	392 (35.3%)	289 (40.5%)	0.025
Biochemical and sarcopenia diagnostic indicators			
Albumin (g/L)	39.88 ± 3.43	39.56 ± 3.16	0.045
ALT (U/L)	20 (14–30)	18 (13–28.2)	0.039
AST (U/L)	15 (12–20)	15 (12–21)	0.147
Total cholesterol (mmol/L)	4.26 ± 1.09	4.56 ± 1.10	< 0.001
Serum creatinine (*μ*mol/L)	74.1 (63.5–90.4)	55.3 (46.7–68.1)	< 0.001
eGFR (mL/min/1.73 m^2^)	94.25 (76.1–103.33)	94.7 (79.77–103.8)	0.368
Fasting glucose (mmol/L)	7.13 (5.89–9.27)	7.23 (5.82–9.13)	0.922
HbA1c (%)	8.58 ± 1.94	8.58 ± 1.84	0.998
FT3 (pmol/L)	3.96 ± 0.63	3.81 ± 0.56	< 0.001
FT4 (pmol/L)	12.67 ± 1.41	12.48 ± 1.47	0.006
TSH (mIU/L)	1.52 (1.04–2.35)	1.97 (1.26–2.76)	< 0.001
ASMI (kg/m^2^)	7.68 ± 0.68	6.36 ± 0.64	< 0.001
Handgrip strength (kg)	33.22 ± 7.95	19.94 ± 5.19	< 0.001
6‐m walk test (m/s)	1.23 ± 0.19	1.16 ± 0.20	< 0.001

*Note:* Data are presented as mean ± SD, median (IQR), or *n* (%). *p* values are for comparisons between men and women.

Abbreviations: ALT, alanine aminotransferase; ASMI, appendicular skeletal muscle mass index; AST, aspartate aminotransferase; DPP‐4i, dipeptidyl peptidase‐4 inhibitors; eGFR, estimated glomerular filtration rate; GLP‐1 RAs, glucagon‐like peptide‐1 receptor agonists; HbA1c, glycated hemoglobin; SGLT2i, sodium–glucose cotransporter‐2 inhibitors.

### 3.2. Associations of Thyroid Hormones With Muscle Parameters

In linear regression analyses, higher FT3 was positively associated with ASMI in the unadjusted and partially adjusted models, but this attenuated following full adjustment (Table 2). FT3 remained significantly associated with handgrip strength after adjustment (Table 3), while its association with gait speed weakened and lost significance (Table 4). In contrast, FT4 was inversely associated with ASMI and positively associated with gait speed in women, whereas TSH was positively associated with ASMI but showed no consistent association with strength or performance. A significant sex interaction was observed for FT3 on handgrip strength (*p* = 0.006), but not for ASMI or gait speed. Furthermore, ROC analysis demonstrated that FT3 had the strongest discriminatory ability for sarcopenia among the thyroid hormones (AUROC = 0.633), whereas FT4 and TSH showed no discriminative value (AUROC = 0.50). For the sarcopenia components, FT3 achieved AUROCs of 0.561 for low muscle mass, 0.586 for low strength, and 0.628 for low gait speed (Figures 1a, 1b, and 1c).

**Table 2:** Linear association between thyroid hormone levels and ASMI.

	**Model (crude)**		**Model 1**		**Model 2**	
	**β**	**p** **value**	**β**	**p** **value**	**β**	**p** **value**
Total						
FT3	0.23	< 0.001	0.07	0.011	0.05	0.072
FT4	−0.01	0.718	−0.04	< 0.001	−0.03	0.002
TSH	−0.02	0.139	0.04	<0.001	0.04	<0.001
Male						
FT3	0.12	<0.001	0.08	0.018	0.04	0.183
FT4	−0.04	0.012	−0.04	0.002	−0.04	0.012
TSH	0.03	0.016	0.04	0.003	0.04	0.014
Female						
FT3	0.06	0.180	0.04	0.372	0.04	0.339
FT4	−0.03	0.054	−0.03	0.050	−0.03	0.046
TSH	0.04	0.015	0.05	0.003	0.05	0.003

*Note:* Model 1: adjusted for age, sex, diabetic nephropathy, smoking status, and alcohol consumption. Model 2: additionally adjusted for HbA1c. Interaction *p* values for sex: FT3 = 0.397, FT4 = 0.646, and TSH = 0.708.

**Table 3:** Linear association between thyroid hormone levels and handgrip strength.

	**Model (crude)**		**Model 1**		**Model 2**	
	**β**	**p** **value**	**β**	**p** **value**	**β**	**p** **value**
Total						
FT3 ^∗^	3.05	< 0.001	0.60	0.019	0.55	0.036
FT4	0.37	0.018	−0.05	0.653	−0.03	0.784
TSH	−0.66	< 0.001	0.15	0.145	0.14	0.182
Male						
FT3	2.26	< 0.001	0.75	0.035	0.70	0.055
FT4	0.04	0.797	−0.13	0.388	−0.11	0.469
TSH	−0.16	0.328	0.11	0.457	0.09	0.539
Female						
FT3	0.74	0.033	0.05	0.886	0.01	0.987
FT4	0.13	0.334	0.07	0.575	0.08	0.522
TSH	0.03	0.845	0.21	0.073	0.21	0.079

*Note:* Model 1: adjusted for age, sex, smoking status, alcohol consumption, diabetic nephropathy, and BMI. Model 2: additionally adjusted for HbA1c. Interaction *p* values for sex: FT3 = 0.006, FT4 = 0.538, and TSH = 0.237.

**Table 4:** Linear association between thyroid hormone levels and gait speed.

	**Model (crude)**		**Model 1**		**Model 2**	
	**β**	**p** **value**	**β**	**p** **value**	**β**	**p** **value**
Total						
FT3	0.06	< 0.001	0.02	0.033	0.01	0.080
FT4	0.01	0.095	0.00	0.952	0.00	0.843
TSH	−0.01	< 0.001	−0.00	0.208	−0.00	0.142
Male						
FT3	0.05	< 0.001	0.02	0.060	0.01	0.118
FT4	−0.00	0.740	−0.01	0.094	−0.01	0.141
TSH	−0.01	0.041	−0.00	0.533	−0.00	0.399
Female						
FT3	0.05	0.001	0.01	0.268	0.01	0.374
FT4	0.01	0.025	0.01	0.055	0.01	0.038
TSH	−0.01	0.008	−0.01	0.299	−0.01	0.266

*Note:* Model 1: adjusted for age, sex, smoking status, alcohol consumption, diabetic nephropathy, and BMI. Model 2: additionally adjusted for HbA1c. Interaction *p* values for sex: FT3 = 0.858, FT4 = 0.016, and TSH = 0.765.

Figure 1ROC analysis of thyroid hormones for sarcopenia and its components. (a) Assessment of low skeletal muscle mass: AUC values: FT3 = 0.561, FT4 = 0.538, and TSH = 0.512. (b) Assessment of low muscle strength: AUC values: FT3 = 0.586, FT4 = 0.525, and TSH = 0.522. (c) Assessment of low physical performance: AUC values: FT3 = 0.628, FT4 = 0.523, and TSH = 0.572. (d) Diagnosis of sarcopenia: AUC values: FT3 = 0.633, FT4 = 0.501, and TSH = 0.500.

**Figure:**
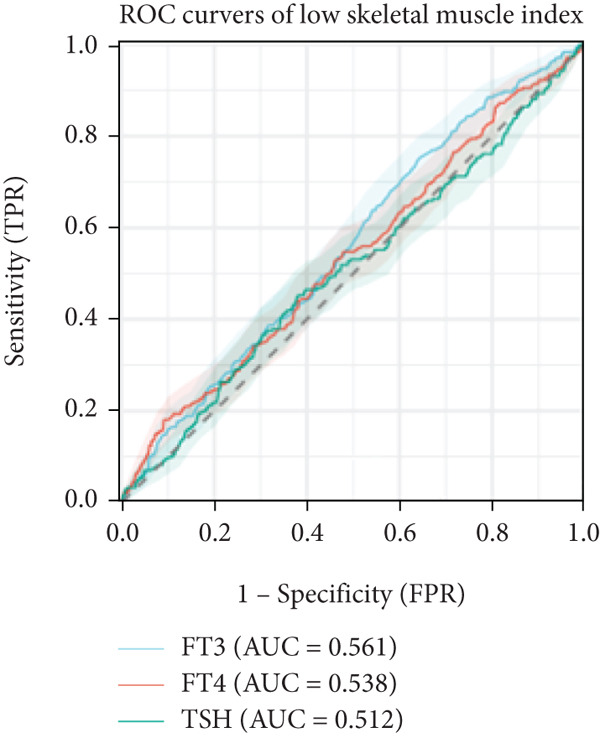
(a)

**Figure:**
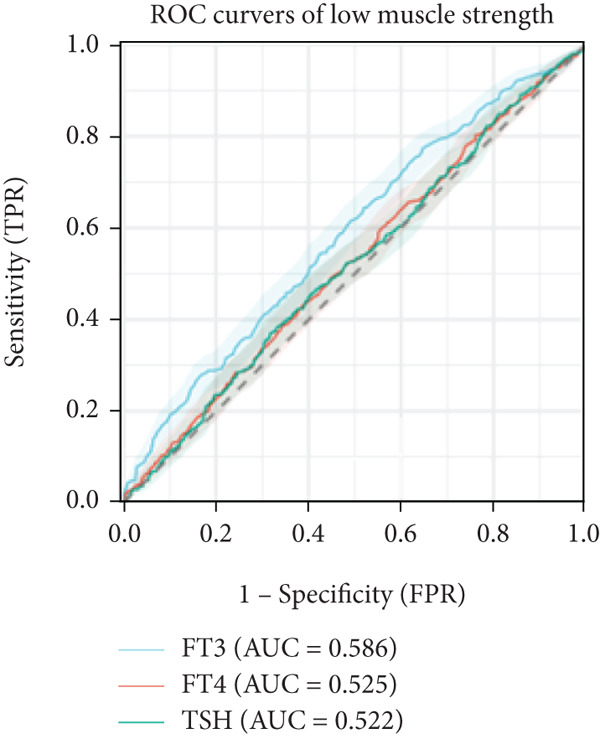
(b)

**Figure:**
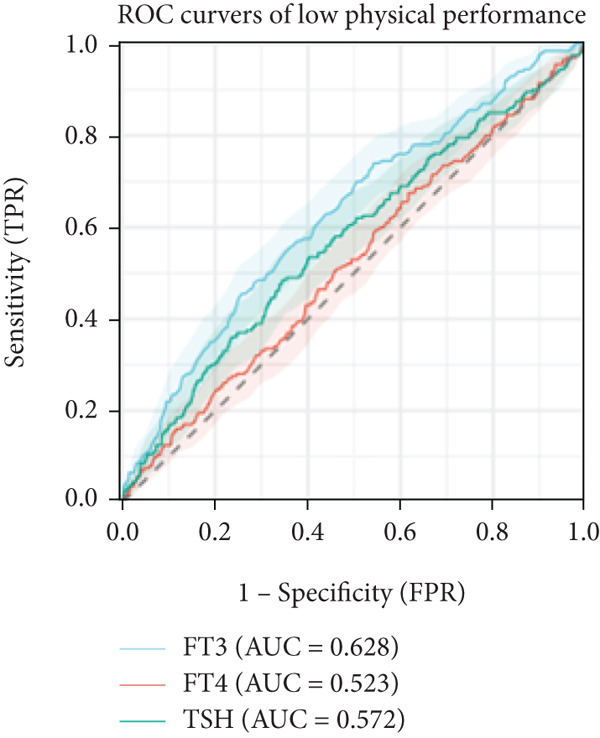
(c)

**Figure:**
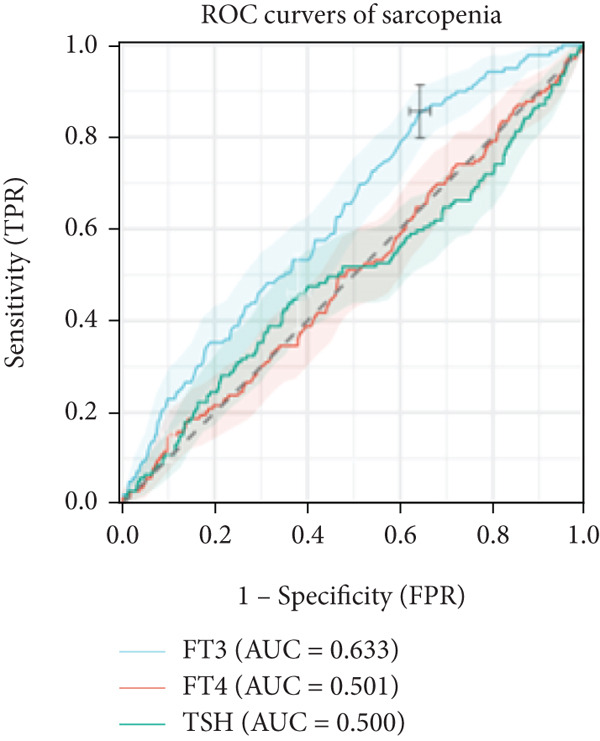
(d)

### 3.3. Prevalence of Sarcopenia Across FT3 Quartiles

When stratified by FT3 quartiles, the prevalence of sarcopenia and its components decreased significantly with increasing FT3 levels in the total population (all *p* < 0.004). Among men, all four outcomes (low muscle mass, low strength, low gait speed, and sarcopenia) significantly decreased (all *p* < 0.022). Among women, significant trends were observed for low gait speed and sarcopenia, while the associations between low muscle mass and low strength were borderline (Table 5). ROC analysis further identified an optimal FT3 cutoff of 3.62 pmol/L for sarcopenia detection, corresponding to a sensitivity of 85.6% and a specificity of 35.7% (Figure 1d).

**Table 5:** Prevalence of sarcopenia and its components across FT3 quartiles.

	**Low ASMI**	**Low muscle strength**	**Low physical performance**	**Sarcopenia**
Total	243	513	200	139
FT3Q1	16.16 (12.93–19.83)	35.34 (30.99–39.88)	17.67 (14.31–21.45)	11.64 (8.86–4.91)
FT3Q2	13.36 (10.35–16.86)	30.51 (26.28–35.00)	11.36 (8.58–14.66)	8.24 (5.87–11.18)
FT3Q3	14.75 (11.64–18.32)	25.6 (21.67–29.83)	8.03 (5.71–10.89)	7.38 (5.16–10.15)
FT3Q4	8.91 (6.44–11.93)	20.94 (17.26–25.00)	6.68 (4.55–9.40)	3.12 (1.71–5.18)
*Z* score	−2.85	−5.11	−5.57	−4.75
*p* for trend	0.004	< 0.001	< 0.001	< 0.001
Male	147	269	91	81
FT3Q1	17.53 (13.04–22.81)	35.06 (29.17–41.31)	13.55 (9.57–18.41)	13.15 (9.23–7.96)
FT3Q2	11.16 (7.54–15.71)	25.1 (19.86–30.94)	9.16 (5.90–13.43)	6.37 (3.69–10.15)
FT3Q3	15.75 (11.77–20.45)	19.52 (15.13–24.54)	6.51 (3.96–9.97)	7.19 (4.51–10.78)
FT3Q4	9.18 (6.23–12.91)	19.3 (15.10–24.09)	4.75 (2.68–7.71)	3.48 (1.75–6.14)
*Z* score	−2.30	−4.50	−3.92	−4.02
*p* for trend	0.022	< 0.001	< 0.001	< 0.001
Female	96	244	109	58
FT3Q1	14.55 (10.11–20.01)	35.68 (29.25–42.51)	22.54 (17.11–28.74)	9.86 (6.21–14.68)
FT3Q2	16.16 (11.32–22.04)	37.37 (30.61–44.51)	14.14 (9.61–19.79)	10.61 (6.69–5.75)
FT3Q3	13.02 (8.34–19.04)	36.09 (28.86–43.82)	10.65 (6.44–16.31)	7.69 (4.16–12.79)
FT3Q4	8.27 (4.2–14.32)	24.81 (17.74–33.04)	11.28 (6.45–17.92)	2.26 (4.68–6.45)
*Z* score	−1.70	−1.81	−3.25	−2.52
*p* for trend	0.089	0.070	0.001	0.012

*Note:* Data are presented as *n* (%). *p* values were obtained from *χ* ^2^ tests for trend across quartiles. Sarcopenia was defined according to AWGS 2019 criteria.

### 3.4. Associations of FT3 With Sarcopenia Risk and Model Performance

In logistic regression, higher FT3 (continuous variable) was independently associated with lower sarcopenia odds in the total population (Model 2: OR = 0.68; 95% CI, 0.48–0.96; *p* = 0.028; (Table 6). Similar patterns were observed in men (OR = 0.65, *p* = 0.055) and women (OR = 0.78, *p* = 0.403), although the association was not significant in the sex‐stratified models. When categorized, low FT3 was associated with higher sarcopenia risk (total population: OR = 2.26; 95% CI, 1.36–3.78; *p* = 0.002), with stronger associations in women (OR = 3.29; 95% CI, 1.24–8.69; *p* = 0.016) than in men (OR = 1.83, *p* = 0.056). Notably, no significant FT3 × sex interaction was detected (Table 6).

**Table 6:** Association between FT3 levels and sarcopenia risk in T2DM.

	**Total**		**Male**		**Female**	
**FT3**	OR (95% CI)	*p* value	OR (95% CI)	*p* value	OR (95% CI)	*p* value
Model (crude)	0.43	< 0.001	0.38	< 0.001	0.55	0.020
	(0.32–0.59)		(0.26–0.56)		(0.33–0.91)	
Model 1	0.63	0.008	0.63	0.032	0.67	0.153
	(0.45–0.89)		(0.41–0.96)		(0.38–1.16)	
Model 2	0.68	0.028	0.652	0.055	0.78	0.403
	(0.48–0.96)		(0.42–1.01)		(0.44–1.39)	
**Low FT3**						
Model (crude)	3.31	< 0.001	2.93	< 0.001	4.36	0.002
	(2.04–5.37)		(1.65–5.20)		(1.72–11.08)	
Model 1	2.32	0.001	1.88	0.043	3.48	0.011
	(1.40–3.86)		(1.02–3.48)		(1.33–9.09)	
Model 2	2.26	0.002	1.83	0.056	3.29	0.016
	(1.36–3.78)		(0.99–3.39)		(1.24–8.69)	

*Note:* Model 1: adjusted for age, sex, BMI, and duration of diabetes. Model 2: additionally adjusted for HbA1c, diabetic nephropathy, insulin use, and *α*‐glucosidase inhibitor use. Interaction *p* values for sex: FT3 × sex = 0.559 and low FT3 × sex = 0.375.

To further assess predictive performance, we compared confounder‐adjusted models with and without thyroid hormones. Adding FT3 significantly improved discrimination, increasing AUROC from 0.814 to 0.821 (*Δ*AUROC = 0.007; 95% CI, 0.000–0.014; *p* = 0.04; (Table 7), whereas FT4 and TSH did not significantly alter AUROC. Reclassification analyses confirmed that continuous FT3 provided a modest incremental value with significant improvement at the 25th percentile risk threshold (NRI = 0.032; *p* = 0.009), although the overall category‐free NRI and IDI were not statistically significant (Table S1).

**Table 7:** Predictive discrimination of thyroid markers in multivariable‐adjusted logistic models.

**Marker**	**AUC (95% CI) (base model)**	**AUC (95% CI) (with marker)**	***Δ*AUC (95% CI)**	**p** **value**
FT3	0.814 (0.780–0.847)	0.821 (0.788–0.852)	0.007 (0.000–0.014)	0.04
FT4	0.814 (0.780–0.847)	0.814 (0.780–0.847)	0.000 (−0.002–0.002)	0.998
TSH	0.814 (0.780–0.847)	0.815 (0.782–0.847)	0.001 (−0.001–0.002)	0.268

*Note:* Baseline model: adjusted for age, sex, BMI, duration of diabetes, diabetic nephropathy, insulin use, *α*‐glucosidase inhibitor use, and HbA1c. New models: baseline + FT3, baseline + FT4, or baseline + TSH. Estimates were derived from logistic regression models; 95% confidence intervals and *p* values were obtained using 500 bootstrap resamples.

Abbreviations: *Δ*AUC, change in AUC after adding the marker to the baseline model; AUC, area under the receiver operating characteristic curve.

### 3.5. Clinical Characteristics and Risk of Sarcopenia by FT3 Cutoff

Stratified by the ROC‐derived FT3 cutoff (3.62 pmol/L), patients in the low‐FT3 group were older, had lower BMI, worse renal function (higher creatinine and lower eGFR), lower serum albumin, higher HbA1c, and poorer muscle measures (ASMI, handgrip strength, and gait speed). Sarcopenia prevalence was also markedly higher in the low FT3 group than in the high FT3 group (9.9% vs. 3.2%; (Table 8).

**Table 8:** Demographic and clinical characteristics of subjects categorized by FT3 cutoff.

	**Hight FT3 (622)**	**Low FT3 (1201)**	**p** **value**
Demographic variables			
Age (years)	60.30 (8.46)	64.38 (9.29)	< 0.001
Sex			
Male	426 (68.5%)	684 (57.0%)	< 0.001
Female	196 (31.5%)	517 (43.0%)	
BMI (kg/m^2^)	25.14 (3.04)	24.65 (3.15)	0.002
Smoking status, *n* (%)			0.005
Current	172 (27.7%)	252 (21.0%)	
Past	17 (2.7%)	44 (3.7%)	
Alcohol consumption status	102 (16.4%)	156 (13.0%)	0.048
Duration of diabetes (months)	108 (48–168)	120 (48–192)	0.012
Diabetic nephropathy	178 (28.6%)	469 (39.1%)	< 0.001
Hypertension	359 (57.7%)	744 (62.0%)	0.08
Sarcopenia	20 (3.2%)	119 (9.9%)	< 0.001
Diabetic medication usage			
Metformin	253 (40.7%)	466 (38.8%)	0.438
Pioglitazone	13 (2.1%)	30 (2.5%)	0.586
SGLT2	57 (9.2%)	161 (13.4%)	0.008
*α*‐Glucosidase inhibitors	157 (25.2%)	316 (26.3%)	0.621
Sulfonylureas	147 (23.6%)	293 (24.4%)	0.718
Repaglinide	16 (2.6%)	57 (4.8%)	0.025
GLP‐1 RAs	25 (4.0%)	55 (4.6%)	0.58
DPP‐4i	79 (12.7%)	155 (12.9%)	0.901
Insulin	220 (35.4%)	461 (38.4%)	0.207
Clinical indicators			
Albumin	40.73 (3.04)	39.26 (3.36)	< 0.001
ALT	22 (15.8–33)	18 (13–27)	< 0.001
AST	16 (12–22)	14.9 (12–20)	< 0.001
Total cholesterol	4.38 (1.07)	4.37 (1.12)	0.847
Serum creatinine	65.4 (55–79.7)	68.3 (55.3–85.8)	0.019
eGFR	97.86 (86.37–105.22)	91.98 (73.68–102.3)	< 0.001
Fasting glucose	7.05 (5.95–8.88)	7.28 (5.81–9.44)	0.289
HbA1c %	8.22 (1.66)	8.77 (1.99)	< 0.001
Skeletal muscle index	7.33 (0.89)	7.08 (0.93)	< 0.001
Handgrip strength	30.14 (9.36)	26.93 (9.45)	< 0.001
6‐m walk test	1.23 (0.19)	1.18 (0.20)	< 0.001

*Note:* Data are presented as mean ± SD, median (IQR), or *n* (%). *p* values are for comparisons between groups above and below the FT3 cutoff (3.62 pmol/L).

Abbreviations: ALT, alanine aminotransferase; AST, aspartate aminotransferase; DPP‐4i, dipeptidyl peptidase‐4 inhibitors; eGFR, estimated glomerular filtration rate; GLP‐1 RAs, glucagon‐like peptide‐1 receptor agonists; HbA1c, glycated hemoglobin; SGLT2i, sodium–glucose cotransporter‐2 inhibitors.

In multivariable logistic regression, high FT3 (≥ 3.62 pmol/L) significantly correlated with lower sarcopenia risk in the cohort (Model 2: OR = 0.52; 95% CI, 0.31–0.89; *p* = 0.015; (Table 9). Stratified analyses indicated significance in women (OR = 0.33; 95% CI, 0.12–0.87; *p* = 0.025) but not in men (OR = 0.64; 95% CI, 0.31–1.30; *p* = 0.220; (Table 9). The corresponding forest plots depict these associations: Figure 2a summarizes the overall cohort, and (Figure 2b) displays the sex‐stratified results (men and women analysed side‐by‐side); no significant sex interaction was detected. Reclassification analyses, based on the FT3 cutoff, showed modest incremental values, with significant improvement at the 25th percentile risk threshold (NRI = 0.032, *p* = 0.009) and borderline significance at the 50th percentile, while overall category‐free NRI and IDI were not significant (Table S2).

**Table 9:** FT3 levels and sarcopenia risk in Type 2 diabetes: gender‐specific analysis.

	**Model (crude)**		**Model 1**		**Model 2**	
	**OR (95% CI)**	**p** **value**	**OR (95% CI)**	**p** **value**	**OR (95% CI)**	**p** **value**
Total						
Low FT3	1.00	/	1.00	/	1.00	/
	/		/		/	
High FT3	0.30	< 0.001	0.46	0.003	0.52	0.015
	(0.19–0.49)		(0.27–0.76)		(0.31–0.89)	
Male						
Low FT3	1.00	/	1.00	/	1.00	/
	/		/		/	
High FT3	0.34	< 0.001	0.56	0.068	0.72	0.331
	(0.19–0.61)		(0.30–1.04)		(0.38–1.39)	
Female						
Low FT3	1.00	/	1.00	/	1.00	/
	/		/		/	
High FT3	0.23	0.002	0.31	0.017	0.33	0.025
	(0.09–0.58)		(0.12–0.82)		(0.12–0.87)	

*Note:* Model 1: adjusted for age, sex, BMI, smoking status, alcohol consumption, diabetic nephropathy, and duration of diabetes. Model 2: additionally adjusted for SGLT2 inhibitors, repaglinide, albumin, HbA1c, ALT, and AST.

Figure 2Forest plots showing the clinical characteristics associated with high FT3 levels in patients with T2DM. (a) Overall population. (b) Sex‐stratified subgroup analysis (males and females). Odds ratios (OR) and 95% confidence intervals (CI) were calculated to evaluate the associations. Statistical significance is indicated by *p* values. Sex‐stratified results are shown for illustrative purposes; no significant interaction by sex swas detected.

**Figure:**
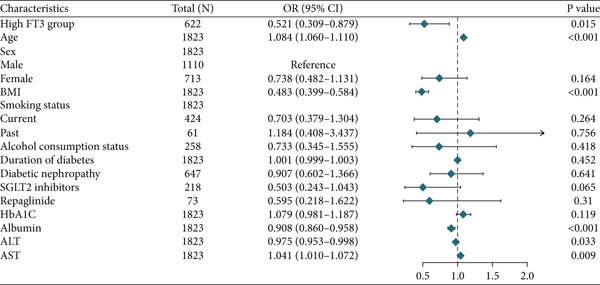
(a)

**Figure:**
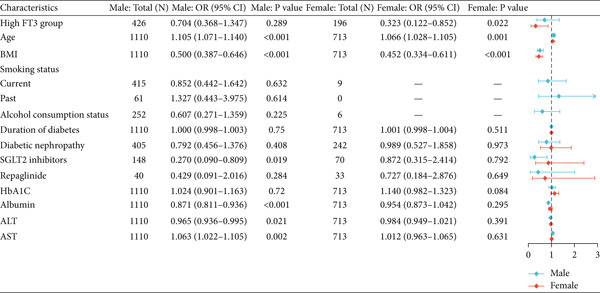
(b)

## 4. Discussion

This study examined the relationship between thyroid hormone levels and sarcopenia in 1823 patients with T2DM. FT3 emerged as the most informative thyroid marker for sarcopenia and showed the strongest discriminatory value on ROC analysis (AUROC = 0.633), with an exploratory cutoff of 3.62 pmol/L (sensitivity, 72.6% and specificity, 52.1; (Figure 1d), requiring external validation. In the multivariate models, FT3 remained positively associated with handgrip strength, while associations with ASMI and gait speed attenuated after full adjustment (Tables 2, 3, and 4). Higher FT3 levels were independently associated with lower sarcopenia odds, with a trend toward stronger effects in women, although no significant FT3 × sex interactions were detected (Table 6 and Figure 2a,b). Beyond risk association, adding FT3 to a confounder‐adjusted model yielded a statistically significant but modest improvement in discrimination (*Δ*AUROC = 0.007, *p* = 0.04; (Table 7); FT4 and TSH showed no benefit. Reclassification metrics showed a significant NRI at the 25th percentile risk threshold (Table S1). Collectively, FT3 may be a potentially relevant biomarker for sarcopenia risk stratification in patients with T2DM, providing a preliminary incremental predictive value over conventional factors (Table S2).

Our findings extend those of previous studies linking thyroid status to muscle health. Experimental and clinical evidence shows that thyroid hormones regulate muscle gene expression, metabolism, and mitochondrial function, thereby shaping skeletal muscle aging [18]. Consistent with Sheng et al. [19], we observed beneficial associations between FT3 and muscle mass/function indices; our results in a larger T2DM cohort align with reports in euthyroid diabetic populations [20, 21]. Notably, quartile analyses showed a stepwise decline in the prevalence of low muscle mass, strength, gait speed, and sarcopenia with increasing FT3 levels (Table 5), suggesting a possible dose–response relationship. Conversely, the ELSA‐Brasil study did not detect associations between thyroid function and sarcopenia in a general population sample [13], possibly owing to differences in population characteristics (community‐dwelling vs. T2DM patients) or methodological approaches, including diagnostic criteria. By incorporating sex stratification and pharmacotherapy covariates and formally testing incremental predictions, our study adds to the literature and provides preliminary evidence regarding the potential clinical utility of FT3 in a high‐risk metabolic context.

Several biological pathways may underlie the FT3–sarcopenia link in T2DM. Hyperglycemia‐related alterations in deiodinase activity, notably reduced type II iodothyronine deiodinase (DIO2), can limit peripheral T4‐to‐T3 conversion and blunt T3‐mediated anabolic signalling in the muscles [24–27]. Lower FT3 levels may reduce GLUT4 expression and impair insulin‐stimulated glucose uptake, thereby promoting muscle atrophy [24, 28, 29]. T3 is also integral to mitochondrial biogenesis and oxidative capacity via PGC‐1*α*–dependent transcriptional programs; diminished T3 signalling can exacerbate mitochondrial dysfunction and oxidative stress [30, 31], contributing to type I fiber vulnerability observed in T2DM [32]. Additionally, T3 influences fibre‐type specification and remodelling through AMPK–PGC‐1*α* pathways and mitochondrial biogenesis; reduced T3 may shift fibers toward glycolytic phenotypes and impair regeneration [33–38]. These mechanisms of insulin resistance, mitochondrial dysfunction, and fiber type remodeling provide a coherent biological framework for epidemiological observations.

Clinically, our data suggest that FT3 level may assist in early sarcopenia risk identification in patients with T2DM and complement conventional risk factors in predictive models. The ROC‐derived cutoff (3.62 pmol/L; (Figure 1d) may serve as a preliminary threshold but requires external validation before clinical adoption. Incremental analyses demonstrated statistically significant but modest gains in discrimination and reclassification (*Δ*AUROC = 0.007; NRI at the 25th percentile; (Table 7); (Tables S1 and S2), supporting potential value for risk enrichment in research settings rather than immediate clinical use. A trend toward stronger associations was observed in women (Table 6) and (Figure 2b), although the interaction by sex was nonsignificant, suggesting a possible sex‐specific susceptibility that merits further study. From a practical perspective, FT3 measurement is inexpensive and widely available, which may facilitate its consideration in future risk stratification strategies once prospective validation is achieved.

This study has several limitations. First, its cross‐sectional design limited its ability to infer causality. Although residual confounding cannot be fully ruled out, we minimized bias by including clinically relevant covariates identified from the univariate screening (Table 9). Information on nutrition and physical activity was unavailable, and thyroid function was assessed at a single time point, which may not fully reflect long‐term hormone status. Muscle mass was measured using BIA rather than DXA; however, BIA was validated against DXA and is recommended by the AWGS 2019 for both clinical and epidemiological applications. Finally, the participants were recruited from tertiary hospitals in China, and the findings may not be generalisable to other populations; however, the large sample size and standardized measurements enhance the robustness of our conclusions. Future research should include multicentre longitudinal cohorts, external validation of FT3‐based prediction models with clinically meaningful thresholds, and mechanistic studies on deiodinase activity, mitochondrial bioenergetics, and muscle fiber remodeling in T2DM.

## 5. Conclusion

Lower FT3 levels are associated with higher sarcopenia risk in T2DM, with a trend toward stronger associations in women. FT3 showed the strongest discriminatory performance among the thyroid hormones and may be a useful biomarker for early risk identification, pending external validation. Incorporating FT3 into the confounder‐adjusted models yielded statistically significant but modest improvements in model performance, indicating a preliminary incremental predictive value beyond that of conventional factors. Prospective multicentre studies are warranted to confirm these findings, establish temporal relationships, and evaluate FT3′s predictive and clinical utility in sarcopenia risk stratification.

## Conflicts of Interest

The authors declare no conflicts of interest.

## Funding

This work was supported by the National Key Research and Development Program of China, (Grant No. 2018YFC1314100), and the Taihu Lake Talent Plan of Wuxi, No. Y20242106.

## Supporting information


**Supporting Information** Additional supporting information can be found online in the Supporting Information section. Tables S1 and S2: The reclassification analyses (NRI and IDI) for thyroid function indicators.

## Data Availability

The data used to support the findings of this study are not publicly available due to patient privacy and ethical restrictions but are available from the corresponding author upon reasonable request.
