# Correction to “Associations of Free Triiodothyronine With Sarcopenia Among Patients With Type 2 Diabetes: Cross‐Sectional and Sex‐Stratified Analyses”

**DOI:** 10.1155/jdr/9803941

**Published:** 2026-05-27

J. Zhao, M‐T. Zhang, D. Yu, Z‐Y. Shao, D‐S. Chen, J. Zhu, “Associations of Free Triiodothyronine With Sarcopenia Among Patients With Type 2 Diabetes: Cross‐Sectional and Sex‐Stratified Analyses,” *Journal of Diabetes Research*, 2026, 3134701, 10.1155/jdr/3134701

In the article, there are errors in the funding statement. The correct funding statement is shown below:

This work was supported by the National Key Research and Development Program of China, (Grant No. 2018YFC1314100) and the Wuxi Science and Technology Development Fund Project “Light of the Taihu Lake” (Y20242106).

We apologize for these errors.

