# Supplementary material for: Associations of Free Triiodothyronine With Sarcopenia Among Patients With Type 2 Diabetes: Cross‐Sectional and Sex‐Stratified Analyses
Source: J Diabetes Res. 2026 Feb 8;2026:3134701. doi: 10.1155/jdr/3134701 (PMC12883552; doi:10.1155/jdr/3134701)
Supplement: Supplementary file 1 — Supporting Information Additional supporting information can be found online in the Supporting Information section. Tables S1 and S2: The reclassification analyses (NRI and IDI) for thyroid function indicators. [file JDR-2026-3134701-s001.docx]

| Supplementary Table S1. Incremental reclassification value of FT3 in confounder-adjusted logistic regression models | | | | |
| --- | --- | --- | --- | --- |
| **Threshold setting** | **Metric** | **Estimate** | **95% CI** | **P value** |
| **Categoryless** | NRI | 0.106 | –0.072–0.272 | 0.248 |
|  | IDI | 0.002 | –0.003–0.008 | 0.43 |
| **25th percentile (0.011)** | NRI | 0.011 | 0.002–0.020 | 0.025 |
| **50th percentile (0.031)** | NRI | 0.01 | –0.004–0.029 | 0.217 |

Abbreviations: NRI, net reclassification improvement; IDI, integrated discrimination improvement;

Baseline model: adjusted for age, sex, BMI, and duration of diabetes, diabetic nephropathy, insulin use, α-glucosidase inhibitor use, and HbA1c.

New model: baseline + FT3 (continuous variable)

Estimates were derived from logistic regression models; 95% confidence intervals and p values were obtained using 500 bootstrap resamples.

| Supplementary Table S2. Reclassification performance of FT3 in ROC cut-off–based logistic regression models | | | | |
| --- | --- | --- | --- | --- |
| **Threshold setting** | **Metric** | **Estimate** | **95% CI** | **P value** |
| **Categoryless** | IDI | 0.002 | −0.004 to 0.007 | 0.421 |
|  | NRI | 0.013 | −0.002 to 0.036 | 0.082 |
| **25th percentile (0.011)** | IDI | 0.002 | −0.004 to 0.007 | 0.414 |
|  | NRI | 0.032 | 0.017 to 0.050 | 0.009 |
| **50th percentile (0.031)** | IDI | 0.002 | −0.004 to 0.007 | 0.391 |
|  | NRI | 0.013 | −0.001 to 0.026 | 0.062 |

NRI, net reclassification improvement; IDI, integrated discrimination improvement;

Base model: adjusted for age, sex, BMI, duration of diabetes, diabetic nephropathy, insulin use, α-glucosidase inhibitor use, and HbA1c.

New model: base model + FT3 (dichotomized at the ROC-derived cutoff of 3.62 pmol/L).
Estimates were derived from logistic regression models; 95% confidence intervals and p values were obtained using 500 bootstrap resamples.
